# Supplementary material for: In Silico Assessment of Potential Geroprotectors: From Separate Endpoints to Complex Pharmacotherapeutic Effects
Source: Int J Mol Sci. 2025 Sep 11;26(18):8858. doi: 10.3390/ijms26188858 (PMC12470084; doi:10.3390/ijms26188858)
Supplement: Supplementary file 1 [file ijms-26-08858-s001.zip › ijms-3835194-supplementary.pdf]

Supplementary Materials

Table S1. The complete predicted activity spectra for the reference compounds.

Compound	Pa and Pi values
Acetylsalicylic acid	0.596 0.023 Transcription factor STAT1 inhibitor<br>0.549 0.032 Transcription factor NF kappa B inhibitor<br>0.523 0.015 SMAD3 inhibitor<br>0.456 0.074 Autophagy inducer<br>0.417 0.033 Transcription factor NF kappa B1 inhibitor<br>0.414 0.016 Transcription factor STAT3 inhibitor<br>0.404 0.027 Free radical scavenger<br>0.404 0.019 Transcription factor STAT inhibitor<br>0.401 0.015 NF-E2-related factor 2 stimulant<br>0.379 0.038 Antioxidant<br>0.365 0.018 Antimutagenic<br>0.356 0.020 Histone deacetylase SIRT6 inhibitor<br>0.354 0.015 Transcription factor STAT5 inhibitor<br>0.324 0.012 Neurotrophic factor enhancer<br>0.311 0.059 Hypoxia-inducible factor 1 alpha inhibitor<br>0.275 0.014 Chelator<br>0.264 0.023 Lipid peroxidase inhibitor<br>0.255 0.242 Nuclear receptor coactivator 3 inhibitor<br>0.234 0.130 Mitochondrial electron transport inhibitor<br>0.214 0.078 Antiamyloidogenic<br>0.156 0.319 Nuclear receptor coactivator 1 inhibitor<br>0.155 0.016 Histone acetyltransferase KAT5 inhibitor<br>0.153 0.006 Chelator. Iron<br>0.151 0.050 Cyclooxygenase 2 inhibitor<br>0.145 0.014 Peroxisome proliferator-activated receptor gamma agonist<br>0.143 0.018 Insulin like growth factor 2 antagonist<br>0.140 0.013 TRKB agonist<br>0.139 0.162 Histone acetyltransferase inhibitor<br>0.119 0.060 Transcription factor STAT6 inhibitor<br>0.115 0.091 Histone acetyltransferase KAT2A inhibitor<br>0.104 0.121 Neurotrophic factor
Atorvastatin	0.219 0.359 Transcription factor STAT1 inhibitor<br>0.201 0.189 Mitochondrial electron transport inhibitor<br>0.181 0.173 Transcription factor NF kappa B inhibitor<br>0.179 0.194 Histone deacetylase SIRT6 inhibitor<br>0.165 0.342 Transcription factor STAT5 inhibitor<br>0.110 0.225 RAGE receptor antagonist
Curcumin	0.852 0.004 Free radical scavenger<br>0.801 0.004 Antioxidant<br>0.775 0.012 Transcription factor NF kappa B inhibitor<br>0.769 0.004 Antimutagenic<br>0.687 0.005 Antiamyloidogenic<br>0.684 0.004 Transcription factor NF kappa B1 inhibitor<br>0.654 0.024 Autophagy inducer

	0.550 0.012 SMAD3 inhibitor<br>0.535 0.005 Transcription factor STAT3 inhibitor<br>0.524 0.007 Transcription factor STAT inhibitor<br>0.407 0.027 Hypoxia-inducible factor 1 alpha inhibitor<br>0.398 0.005 Histone acetyltransferase inhibitor<br>0.386 0.143 Nuclear receptor coactivator 3 inhibitor<br>0.386 0.010 Lipid peroxidase inhibitor<br>0.354 0.008 Chelator<br>0.335 0.030 NF-E2-related factor 2 stimulant<br>0.289 0.071 Mitochondrial electron transport inhibitor<br>0.282 0.003 Insulin like growth factor 2 antagonist<br>0.273 0.089 Transcription factor STAT5 inhibitor<br>0.271 0.096 Nuclear receptor coactivator 1 inhibitor<br>0.260 0.315 Transcription factor STAT1 inhibitor<br>0.258 0.033 Neurotrophic factor enhancer<br>0.214 0.005 Estradiol 17 beta-dehydrogenase inhibitor<br>0.207 0.013 DNA directed RNA polymerase inhibitor<br>0.193 0.031 Cyclooxygenase 2 inhibitor<br>0.181 0.004 Histone deacetylase SIRT2 stimulant<br>0.180 0.004 Chelator. Iron<br>0.155 0.007 Transcription factor RelA inhibitor<br>0.150 0.327 Histone deacetylase SIRT6 inhibitor<br>0.145 0.060 Neurotrophic factor<br>0.134 0.011 Histone acetyltransferase p300 inhibitor<br>0.121 0.046 Histone acetyltransferase KAT5 inhibitor<br>0.110 0.029 Inducible nitric-oxide synthase inhibitor
Dasatinib	0.168 0.294 Nuclear receptor coactivator 1 inhibitor<br>0.161 0.393 Nuclear receptor coactivator 3 inhibitor<br>0.155 0.402 Autophagy inducer<br>0.146 0.121 AMP-activated protein kinase inhibitor<br>0.143 0.139 AMP-activated protein kinase. alpha-1 subunit inhibitor<br>0.135 0.415 Histone deacetylase SIRT6 inhibitor<br>0.122 0.079 AMP-activated protein kinase. beta-1 subunit inhibitor<br>0.110 0.517 Mitochondrial electron transport inhibitor
Fisetin	0.935 0.002 Antimutagenic<br>0.890 0.003 Free radical scavenger<br>0.886 0.004 Antioxidant<br>0.777 0.002 SMAD3 inhibitor<br>0.758 0.014 Autophagy inducer<br>0.662 0.001 TRKB agonist<br>0.634 0.027 Nuclear receptor coactivator 3 inhibitor<br>0.630 0.024 Transcription factor NF kappa B inhibitor<br>0.626 0.006 Hypoxia-inducible factor 1 alpha inhibitor<br>0.625 0.003 NF-E2-related factor 2 stimulant<br>0.608 0.005 Transcription factor NF kappa B1 inhibitor<br>0.540 0.005 Lipid peroxidase inhibitor<br>0.520 0.060 Transcription factor STAT1 inhibitor<br>0.500 0.009 Mitochondrial electron transport inhibitor<br>0.500 0.001 Histone deacetylase SIRT2 stimulant<br>0.479 0.012 Antiamyloidogenic

	0.476 0.010 Transcription factor STAT inhibitor<br>0.458 0.005 Chelator<br>0.430 0.004 Histone deacetylase SIRT1 stimulant<br>0.379 0.004 RAGE receptor antagonist<br>0.330 0.003 Estradiol 17 beta-dehydrogenase inhibitor<br>0.319 0.044 Transcription factor STAT3 inhibitor<br>0.301 0.017 Neurotrophic factor enhancer<br>0.236 0.005 Transcription factor STAT6 inhibitor<br>0.218 0.005 Insulin like growth factor 2 antagonist<br>0.217 0.003 Estradiol 17 beta-dehydrogenase 1 inhibitor<br>0.209 0.225 Transcription factor STAT5 inhibitor<br>0.201 0.005 Transcription factor RelA inhibitor<br>0.199 0.071 Histone acetyltransferase inhibitor<br>0.196 0.231 Nuclear receptor coactivator 1 inhibitor<br>0.194 0.031 Cyclooxygenase 2 inhibitor<br>0.142 0.063 Neurotrophic factor<br>0.136 0.004 Estradiol 17 beta-dehydrogenase 2 inhibitor<br>0.125 0.017 Chelator. Iron<br>0.115 0.559 Histone deacetylase SIRT6 inhibitor<br>0.101 0.084 Histone acetyltransferase KAT5 inhibitor
Metformin	0.430 0.011 Histone deacetylase SIRT6 inhibitor<br>0.283 0.107 Transcription factor NF kappa B1 inhibitor<br>0.265 0.308 Transcription factor STAT1 inhibitor<br>0.238 0.212 SMAD3 inhibitor<br>0.209 0.093 NF-E2-related factor 2 stimulant<br>0.195 0.234 Nuclear receptor coactivator 1 inhibitor<br>0.170 0.012 Inducible nitric-oxide synthase inhibitor<br>0.161 0.056 RAGE receptor antagonist<br>0.158 0.070 Antimutagenic<br>0.148 0.424 Nuclear receptor coactivator 3 inhibitor<br>0.142 0.009 5 Hydroxytryptamine 3 antagonist<br>0.138 0.383 Mitochondrial electron transport inhibitor<br>0.104 0.011 Histone acetyltransferase KAT2B inhibitor<br>0.102 0.112 Histone deacetylase SIRT1 inhibitor
Navitoclax	0.239 0.155 Transcription factor STAT5 inhibitor<br>0.116 0.551 Histone deacetylase SIRT6 inhibitor<br>0.108 0.511 Autophagy inducer
Nicotinamide riboside	0.593 0.035 Autophagy inducer<br>0.469 0.085 Nuclear receptor coactivator 3 inhibitor<br>0.449 0.023 Transcription factor NF kappa B1 inhibitor<br>0.409 0.004 Histone deacetylase SIRT1 stimulant<br>0.339 0.071 Transcription factor NF kappa B inhibitor<br>0.332 0.004 Transcription factor STAT6 inhibitor<br>0.299 0.052 Transcription factor STAT5 inhibitor<br>0.248 0.088 Transcription factor STAT inhibitor<br>0.239 0.123 Mitochondrial electron transport inhibitor<br>0.228 0.233 SMAD3 inhibitor<br>0.194 0.116 Antioxidant<br>0.186 0.024 Histone deacetylase class III inhibitor<br>0.181 0.171 Hypoxia-inducible factor 1 alpha inhibitor<br>0.177 0.099 Neurotrophic factor enhancer

	0.161 0.003 Adenosine deaminase inhibitor<br>0.149 0.331 Histone deacetylase SIRT6 inhibitor<br>0.148 0.009 Histone deacetylase SIRT2 inhibitor<br>0.146 0.147 Free radical scavenger<br>0.145 0.064 Histone deacetylase SIRT1 inhibitor<br>0.145 0.025 DNA directed RNA polymerase inhibitor<br>0.121 0.383 Nuclear receptor coactivator 1 inhibitor
Quercetin	0.958 0.001 Antimutagenic<br>0.924 0.003 Antioxidant<br>0.910 0.003 Free radical scavenger<br>0.827 0.008 Autophagy inducer<br>0.817 0.001 SMAD3 inhibitor<br>0.689 0.018 Transcription factor NF kappa B inhibitor<br>0.661 0.005 Hypoxia-inducible factor 1 alpha inhibitor<br>0.629 0.004 Transcription factor NF kappa B1 inhibitor<br>0.627 0.001 Histone deacetylase SIRT2 stimulant<br>0.606 0.035 Nuclear receptor coactivator 3 inhibitor<br>0.574 0.004 Lipid peroxidase inhibitor<br>0.570 0.004 NF-E2-related factor 2 stimulant<br>0.556 0.001 TRKB agonist<br>0.528 0.004 Chelator<br>0.510 0.010 Antiamyloidogenic<br>0.502 0.072 Transcription factor STAT1 inhibitor<br>0.469 0.013 Mitochondrial electron transport inhibitor<br>0.460 0.011 Transcription factor STAT inhibitor<br>0.454 0.004 Histone deacetylase SIRT1 stimulant<br>0.423 0.003 Estradiol 17 beta-dehydrogenase inhibitor<br>0.373 0.004 RAGE receptor antagonist<br>0.326 0.012 Neurotrophic factor enhancer<br>0.326 0.004 Transcription factor STAT6 inhibitor<br>0.305 0.002 Estradiol 17 beta-dehydrogenase 1 inhibitor<br>0.262 0.073 Transcription factor STAT3 inhibitor<br>0.239 0.004 Transcription factor RelA inhibitor<br>0.213 0.026 Cyclooxygenase 2 inhibitor<br>0.197 0.006 Insulin like growth factor 2 antagonist<br>0.190 0.004 Estradiol 17 beta-dehydrogenase 2 inhibitor<br>0.171 0.109 Histone acetyltransferase inhibitor<br>0.165 0.301 Nuclear receptor coactivator 1 inhibitor<br>0.160 0.358 Transcription factor STAT5 inhibitor<br>0.143 0.009 Chelator. Iron<br>0.124 0.084 Neurotrophic factor<br>0.102 0.663 Histone deacetylase SIRT6 inhibitor
Rapamycin	0.980 0.001 mTOR complex 1 inhibitor<br>0.599 0.027 Transcription factor NF kappa B inhibitor<br>0.536 0.003 Neurotrophic factor<br>0.398 0.017 Fibrosis treatment<br>0.343 0.047 Hypoxia-inducible factor 1 alpha inhibitor<br>0.297 0.066 Mitochondrial electron transport inhibitor<br>0.131 0.454 Autophagy inducer
Resveratrol	0.796 0.011 Autophagy inducer<br>0.775 0.003 Antimutagenic

	0.757 0.004 Free radical scavenger<br>0.738 0.005 Antioxidant<br>0.654 0.005 SMAD3 inhibitor<br>0.639 0.026 Nuclear receptor coactivator 3 inhibitor<br>0.632 0.013 Transcription factor STAT1 inhibitor<br>0.595 0.006 Antiamyloidogenic<br>0.567 0.030 Transcription factor NF kappa B inhibitor<br>0.559 0.001 Histone deacetylase SIRT2 stimulant<br>0.557 0.007 Transcription factor NF kappa B1 inhibitor<br>0.503 0.005 NF-E2-related factor 2 stimulant<br>0.480 0.005 Chelator<br>0.470 0.006 Lipid peroxidase inhibitor<br>0.426 0.004 Histone deacetylase SIRT1 stimulant<br>0.382 0.024 Transcription factor STAT inhibitor<br>0.373 0.026 Transcription factor STAT3 inhibitor<br>0.366 0.040 Hypoxia-inducible factor 1 alpha inhibitor<br>0.349 0.032 Nuclear receptor coactivator 1 inhibitor<br>0.325 0.012 Neurotrophic factor enhancer<br>0.306 0.003 Insulin like growth factor 2 antagonist<br>0.279 0.079 Transcription factor STAT5 inhibitor<br>0.272 0.085 Mitochondrial electron transport inhibitor<br>0.238 0.031 Histone acetyltransferase inhibitor<br>0.221 0.004 Transcription factor RelA inhibitor<br>0.197 0.030 Cyclooxygenase 2 inhibitor<br>0.180 0.035 RAGE receptor antagonist<br>0.173 0.011 Transcription factor STAT6 inhibitor<br>0.172 0.222 Histone deacetylase SIRT6 inhibitor<br>0.162 0.046 Neurotrophic factor<br>0.153 0.006 Chelator. Iron<br>0.152 0.058 Histone deacetylase SIRT1 inhibitor<br>0.152 0.007 Estradiol 17 beta-dehydrogenase inhibitor<br>0.146 0.017 Inducible nitric-oxide synthase inhibitor<br>0.146 0.004 Estradiol 17 beta-dehydrogenase 2 inhibitor<br>0.144 0.022 Histone acetyltransferase KAT5 inhibitor<br>0.102 0.024 TRKB agonist<br>0.102 0.012 Histone acetyltransferase KAT2B inhibitor
Spermidine	0.516 0.054 Autophagy inducer<br>0.507 0.010 Antimutagenic<br>0.489 0.007 Histone deacetylase SIRT6 inhibitor<br>0.476 0.005 Chelator<br>0.448 0.023 Transcription factor NF kappa B1 inhibitor<br>0.399 0.016 NF-E2-related factor 2 stimulant<br>0.384 0.024 Antiamyloidogenic<br>0.351 0.214 Transcription factor STAT1 inhibitor<br>0.348 0.005 Histone deacetylase SIRT1 inhibitor<br>0.324 0.004 Inducible nitric-oxide synthase inhibitor<br>0.307 0.109 SMAD3 inhibitor<br>0.303 0.063 Nuclear receptor coactivator 1 inhibitor<br>0.279 0.067 Transcription factor STAT inhibitor<br>0.271 0.230 Nuclear receptor coactivator 3 inhibitor<br>0.263 0.073 Transcription factor STAT3 inhibitor<br>0.236 0.011 Histone deacetylase class III inhibitor

	0.234 0.125 Transcription factor NF kappa B inhibitor<br>0.224 0.015 RAGE receptor antagonist<br>0.218 0.066 AMP-activated protein kinase. alpha-1 subunit inhibitor<br>0.200 0.069 Histone acetyltransferase inhibitor<br>0.181 0.035 Neurotrophic factor<br>0.180 0.084 AMP-activated protein kinase inhibitor<br>0.163 0.005 Chelator. Iron<br>0.151 0.011 TRKB agonist<br>0.151 0.004 Histone acetyltransferase KAT2B inhibitor<br>0.147 0.024 Transcription factor STAT6 inhibitor<br>0.145 0.356 Mitochondrial electron transport inhibitor<br>0.145 0.143 Neurotrophic factor enhancer<br>0.129 0.175 Free radical scavenger<br>0.121 0.206 Antioxidant<br>0.119 0.016 Histone deacetylase SIRT2 inhibitor<br>0.112 0.006 Myc inhibitor<br>0.104 0.009 Histone deacetylase SIRT3 inhibitor<br>0.102 0.083 Histone acetyltransferase KAT5 inhibitor<br>0.102 0.013 Histone deacetylase SIRT5 inhibitor
Sulforophane	0.883 0.000 NF-E2-related factor 2 stimulant<br>0.391 0.170 Transcription factor STAT1 inhibitor<br>0.369 0.004 Inducible nitric-oxide synthase inhibitor<br>0.345 0.047 Antioxidant<br>0.220 0.075 Antiamyloidogenic<br>0.193 0.154 Histone deacetylase SIRT6 inhibitor<br>0.152 0.434 SMAD3 inhibitor<br>0.148 0.213 Transcription factor STAT3 inhibitor<br>0.143 0.426 Autophagy inducer<br>0.133 0.268 Transcription factor STAT inhibitor<br>0.106 0.214 Free radical scavenger<br>0.104 0.363 Transcription factor NF kappa B1 inhibitor
Torin 2	0.398 0.163 Transcription factor STAT1 inhibitor<br>0.361 0.022 Fibrosis treatment<br>0.303 0.035 AMP-activated protein kinase. alpha-1 subunit inhibitor<br>0.299 0.033 AMP-activated protein kinase inhibitor<br>0.225 0.187 Transcription factor STAT5 inhibitor<br>0.210 0.005 mTOR complex 1 inhibitor<br>0.207 0.298 Nuclear receptor coactivator 3 inhibitor<br>0.193 0.313 SMAD3 inhibitor<br>0.169 0.293 Nuclear receptor coactivator 1 inhibitor<br>0.167 0.009 Phosphatidylinositol 3-kinase inhibitor<br>0.157 0.054 Ribosomal protein S6 kinase inhibitor<br>0.155 0.009 Phosphatidylinositol 3-kinase alpha inhibitor<br>0.151 0.135 Histone acetyltransferase inhibitor<br>0.141 0.289 Transcription factor NF kappa B1 inhibitor<br>0.135 0.445 Autophagy inducer<br>0.128 0.251 Hypoxia-inducible factor 1 alpha inhibitor<br>0.117 0.100 AMP-activated protein kinase. beta-1 subunit inhibitor<br>0.109 0.212 Neurotrophic factor enhancer

	0.107 0.012 Phosphatidylinositol 3-kinase beta inhibitor<br>0.101 0.672 Histone deacetylase SIRT6 inhibitor
Urolithin A	0.831 0.003 Antimutagenic<br>0.800 0.002 SMAD3 inhibitor<br>0.738 0.003 RAGE receptor antagonist<br>0.683 0.017 Nuclear receptor coactivator 3 inhibitor<br>0.680 0.005 Free radical scavenger<br>0.665 0.007 Antioxidant<br>0.619 0.025 Transcription factor NF kappa B inhibitor<br>0.614 0.018 Transcription factor STAT1 inhibitor<br>0.574 0.005 Transcription factor NF kappa B1 inhibitor<br>0.531 0.004 NF-E2-related factor 2 stimulant<br>0.469 0.013 Antiamyloidogenic<br>0.466 0.070 Autophagy inducer<br>0.457 0.004 Neurotrophic factor enhancer<br>0.431 0.023 Hypoxia-inducible factor 1 alpha inhibitor<br>0.346 0.034 Nuclear receptor coactivator 1 inhibitor<br>0.340 0.038 Transcription factor STAT inhibitor<br>0.320 0.052 Mitochondrial electron transport inhibitor<br>0.320 0.044 Transcription factor STAT3 inhibitor<br>0.295 0.004 Estradiol 17 beta-dehydrogenase inhibitor<br>0.282 0.021 Lipid peroxidase inhibitor<br>0.247 0.005 TRKB agonist<br>0.211 0.023 Chelator<br>0.209 0.027 Cyclooxygenase 2 inhibitor<br>0.206 0.005 Transcription factor RelA inhibitor<br>0.189 0.035 Histone deacetylase SIRT1 inhibitor<br>0.177 0.102 Histone acetyltransferase inhibitor<br>0.172 0.325 Transcription factor STAT5 inhibitor<br>0.159 0.013 Insulin like growth factor 2 antagonist<br>0.149 0.043 Histone deacetylase class III inhibitor<br>0.145 0.025 Transcription factor STAT6 inhibitor<br>0.143 0.363 Histone deacetylase SIRT6 inhibitor<br>0.141 0.004 Estradiol 17 beta-dehydrogenase 1 inhibitor<br>0.139 0.005 Histone deacetylase SIRT2 stimulant<br>0.133 0.048 Histone acetyltransferase KAT2A inhibitor<br>0.131 0.004 Estradiol 17 beta-dehydrogenase 3 inhibitor<br>0.117 0.100 AMP-activated protein kinase. beta-1 subunit inhibitor<br>0.114 0.025 Chelator. Iron<br>0.100 0.086 Histone acetyltransferase KAT5 inhibitor
Vildagliptin	0.159 0.278 Histone deacetylase SIRT6 inhibitor<br>0.153 0.430 SMAD3 inhibitor<br>0.130 0.076 Neurotrophic factor<br>0.117 0.480 Mitochondrial electron transport inhibitor
